# Composition and Drivers of Gut Microbial Communities in Arctic-Breeding Shorebirds

**DOI:** 10.3389/fmicb.2019.02258

**Published:** 2019-10-09

**Authors:** Kirsten Grond, Jorge W. Santo Domingo, Richard B. Lanctot, Ari Jumpponen, Rebecca L. Bentzen, Megan L. Boldenow, Stephen C. Brown, Bruce Casler, Jenny A. Cunningham, Andrew C. Doll, Scott Freeman, Brooke L. Hill, Steven J. Kendall, Eunbi Kwon, Joseph R. Liebezeit, Lisa Pirie-Dominix, Jennie Rausch, Brett K. Sandercock

**Affiliations:** ^1^Division of Biology, Kansas State University, Manhattan, KS, United States; ^2^U.S. Environmental Protection Agency, Cincinnati, OH, United States; ^3^Migratory Bird Management, U.S. Fish & Wildlife Service, Anchorage, AK, United States; ^4^Wildlife Conservation Society, Fairbanks, AK, United States; ^5^Department of Biology and Wildlife, University of Alaska Fairbanks, Fairbanks, AK, United States; ^6^Manomet Inc., Saxtons River, VT, United States; ^7^Independent Researcher, Nehalem, OR, United States; ^8^Department of Fisheries and Wildlife Sciences, University of Missouri, Columbia, MO, United States; ^9^Denver Museum of Nature & Science, Denver, CO, United States; ^10^Arctic National Wildlife Refuge, U.S. Fish & Wildlife Service, Fairbanks, AK, United States; ^11^Department of Fish and Wildlife Conservation, Virginia Tech, Blacksburg, VA, United States; ^12^Audubon Society of Portland, Portland, OR, United States; ^13^Environment and Climate Change Canada, Iqaluit, NU, Canada; ^14^Environment and Climate Change Canada, Yellowknife, NT, Canada; ^15^Department of Terrestrial Ecology, Norwegian Institute for Nature Research, Trondheim, Norway

**Keywords:** 16S rRNA gene, breeding site, environment, gut microbiome, host health

## Abstract

Gut microbiota can have important effects on host health, but explanatory factors and pathways that determine gut microbial composition can differ among host lineages. In mammals, host phylogeny is one of the main drivers of gut microbiota, a result of vertical transfer of microbiota during birth. In birds, it is less clear what the drivers might be, but both phylogeny and environmental factors may play a role. We investigated host and environmental factors that underlie variation in gut microbiota composition in eight species of migratory shorebirds. We characterized bacterial communities from 375 fecal samples collected from adults of eight shorebird species captured at a network of nine breeding sites in the Arctic and sub-Arctic ecoregions of North America, by sequencing the V4 region of the bacterial 16S ribosomal RNA gene. Firmicutes (55.4%), Proteobacteria (13.8%), Fusobacteria (10.2%), and Bacteroidetes (8.1%) dominated the gut microbiota of adult shorebirds. Breeding location was the main driver of variation in gut microbiota of breeding shorebirds (*R*^2^ = 11.6%), followed by shorebird host species (*R*^2^ = 1.8%), and sampling year (*R*^2^ = 0.9%), but most variation remained unexplained. Site variation resulted from differences in the core bacterial taxa, whereas rare, low-abundance bacteria drove host species variation. Our study is the first to highlight a greater importance of local environment than phylogeny as a driver of gut microbiota composition in wild, migratory birds under natural conditions.

## Introduction

The gut microbiota is important in maintaining gut homeostasis, and contributions to organismal health have received increasing attention over the past decades. Microorganisms in the gastro-intestinal tract play a major role in nutrient uptake and immune function (Leser and Mølbak, 2009; Hooper et al., 2012). Timing of bacterial recruitment in the gut differs among vertebrate taxa. Mammals acquire their initial gut microbial communities from passage through the birth canal (Leser and Mølbak, 2009), but recruitment routes for birds are less well known (Grond et al., 2018). Shorebirds have precocial chicks and their gut microbiota establish from environmental inocula after hatching (Grond et al., 2017). After the initial establishment, gut microbial communities can be modified by a number of intrinsic and extrinsic factors, including host phylogeny, age, or diet (Ley et al., 2008; Goodrich et al., 2014; Hird et al., 2015).

Evolutionary history based on host phylogeny is often the dominant factor that contributes to gut microbiota composition in mammals, including humans (Ley et al., 2008; Goodrich et al., 2014), although diet has also been identified as an important factor (Muegge et al., 2011; Spor et al., 2011). In a community of Neotropical birds, factors associated with host phylogeny also explained most of the variation in gut microbiota composition, followed closely by ecological variables such as local habitat and foraging location (Hird et al., 2015). Phylogeny was also ranked above ecological drivers in explaining gut microbiota composition in a meta-analysis that included a range of phylogenetically and behaviorally distinct birds (Waite and Taylor, 2014). However, previous findings were based on studies with limited sample sizes, and therefore could potentially underestimate the importance of ecological factors. Indeed, several avian studies have concluded that ecological factors, such as local diet and habitat, strongly affect gut microbiota (Hird et al., 2014; Barbosa et al., 2016; Lewis et al., 2016).

To address the relative influence of phylogeny and environment on gut microbiota, we sampled species varying in phylogenetic relatedness across multiple sites. We investigated the influence of both the environment and host identity on the variation in gut microbiota composition in eight species of shorebirds breeding across a network of sites in the Arctic and sub-Arctic of North America. An investigation of shorebirds is particularly helpful for disentangling the factors that control gut microbiota due to their resolved phylogeny and diversity of life-history traits. Further, the shorebirds sampled in this study breed sympatrically at Arctic breeding sites, thus permitting simultaneous sampling of multiple species within the same environment during one stage of their annual cycle.

The three objectives of our study were to: (1) characterize the bacterial gut microbiota of migratory shorebirds present during the breeding season in the Arctic and sub-Arctic ecoregions; (2) examine which factors explain variation in gut microbiota composition; and (3) assess the relative contribution of host and environmental factors among species or breeding sites on the community and structure of the shorebird gut microbiota. We predicted that gut microbial composition of shorebirds would be predominantly driven by environmental factors, resulting in higher similarity in gut microbiomes in shorebirds breeding at the same site. We collected fecal samples from eight shorebird species at nine breeding sites in Alaska and Canada, and used high-throughput sequencing to characterize their gut microbiota.

## Materials and Methods

### Sample Collection

**FIGURE 1:**
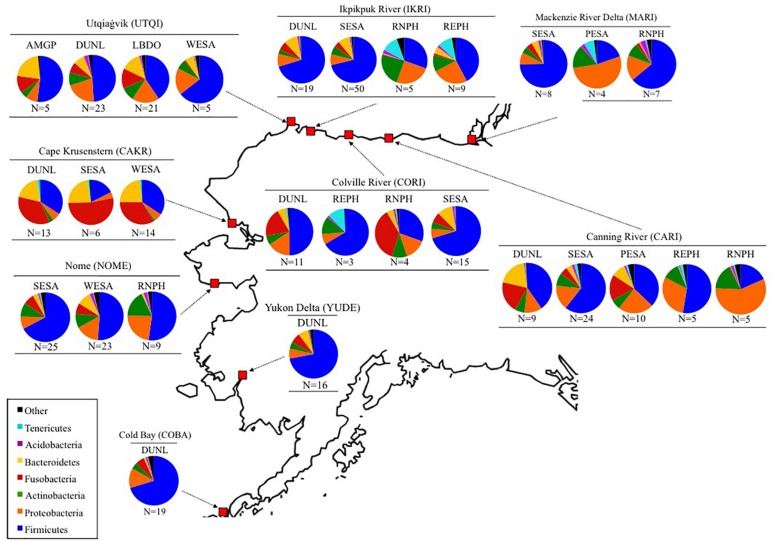
Bacterial communities found in fecal samples of eight shorebird species at nine sites in Alaska and Canada (MacKenzie River Delta) from 2011 to 2014. *N* represents the sample sizes, and bacterial composition is depicted on the Phylum level. Full species names can be found in Table 1.

We sampled shorebirds in collaboration with participating researchers in the Arctic Shorebird Demographics Network (ASDN; Lanctot and Brown, 2014). We collected fecal samples from eight shorebird species at nine sites distributed across 2700 km of the Arctic and sub-Arctic of North America from 2011 to 2014 (Tables 1–4 and Figure 1). Nests were located by using species-specific bird behavior to follow birds to their nests or by dragging ropes to flush incubating birds. Birds were trapped at their nest using walk-in traps and bow nets, and upon capture were placed in a darkened, plastic box for up to 5 min. Collection boxes were sterilized with bleach wipes, and the bottom of the box was lined with a clean sheet of wax paper before reuse. After defecating, birds were banded and biometric measurements were collected. Birds were released within 30 min of capture. Fecal samples were transferred from the wax paper using a sterile tongue depressor to a 1.5 ml sterile Eppendorf tube. To avoid possible contamination, all handling of the wax paper was conducted while wearing sterilized latex gloves. Fecal samples were preserved in 100% ethanol at collection, and stored frozen at −20°C until further microbiome analyses.

**TABLE 1:** Shorebird species investigated in our study.

**Species**	**Scientific name**	**Abbreviation**	**Habitat**
American golden plover	*Pluvialis dominica*	AMGP	T
Long-billed dowitcher	*Limnodromus scolopaceus*	LBDO	M
Pectoral sandpiper	*Calidris melanotos*	PESA	TM
Dunlin	*Calidris alpina*	DUNL	TM
Semipalmated sandpiper	*Calidris pusilla*	SESA	TM
Western sandpiper	*Calidris mauri*	WESA	T
Red phalarope	*Phalaropus fulicarius*	REPH	A
Red-necked phalarope	*Phalaropus lobatus*	RNPH	A

*Habitat categories consist of terrestrial (T), terrestrial/mesic (TM), mesic (M), and aquatic (A).*

**TABLE 2:** Locations and sampling years of field sites in the Arctic Shorebird Demographics Network.

**Site**		**Abbreviation**	**Latitude (°N)**	**Longitude (°W)**	**Years sampled**
Cold Bay	AK, United States	COBA	55.204500	−162.718400	2011
Yukon Delta	AK, United States	YUDE	61.368900	−163.716100	2011
Nome	AK, United States	NOME	64.497934	−165.408204	2011, 2013, 2014
Cape Krusenstern	AK, United States	CAKR	67.417246	−163.874238	2013, 2014
Utqiagvik	AK, United States	UTQI	71.292646	−156.782563	2011
Ikpikpuk River	AK, United States	IKRI	70.814400	−154.405300	2011, 2013
Colville River	AK, United States	CORI	70.384028	−150.806197	2011, 2013
Canning River	AK, United States	CARI	69.945375	−145.098152	2011, 2013
Mackenzie River	NWT, Canada	MARI	68.815927	−137.090836	2011

**TABLE 3:** Sample sizes per site per species after rarefaction.

**Site**	**American golden plover**	**Long-billed dowitcher**	**Pectoral sandpiper**	**Dunlin**	**Semipalmated sandpiper**	**Western sandpiper**	**Red-necked phalarope**	**Red phalarope**
Cold Bay				19				
Yukon Delta				16				
Nome					25	23	9	
Cape Krusenstern				13	6	14	1	
Utqiagvik	5	21		23	2	5	1	1
Ikpikpuk River				19	50		5	9
Colville River			2	11	15	1	4	3
Canning River			10	9	24		5	5
Mackenzie River			4		8		7	

Sampling methods were approved by the Kansas State University Institutional Animal Care and Use Committee, and sampling was conducted under permit numbers 3261 and 3409.

### Molecular Analyses

#### DNA Extraction

Ethanol was removed by centrifuging the fecal samples for 10 min at 10,000 rpm and discarding the supernatant. Our initial cleaning step was repeated twice with 1 ml of RNase/DNase-free molecular grade water (Grond et al., 2014; Ryu et al., 2014). Total DNA was isolated from cleaned fecal samples using the MoBio Power Lyzer/Power Soil kit as per the manufacturer’s instructions (Mo Bio Laboratory, Carlsbad, CA, United States), except for replacing the bead-beating step with 15 min high velocity vortexing. Genomic DNA yields were determined spectrophotometrically (NanoDrop 2000, Thermo Fisher Scientific, Waltham, MA, United States).

#### PCR

16S rRNA gene libraries were generated from DNA extracts using bacterial primers 515F (5′-GTGCCAGCMGCCGCGGTAA-3′) and 806R (5′-GGACTACHVGGTWTCTAAT-3′) targeting the V4 region of the 16S rRNA gene (Caporaso et al., 2012). Primers 806R were uniquely barcoded. We performed PCR reactions in triplicate in a 25 μl reaction volume, using TaqMan^®^ Universal PCR Master Mix (Applied Biosystems, Waltham, MA, United States) and 5 μl of DNA template (5 ng DNA/μl). PCR conditions consisted of 25 cycles of: 15 s at 95°C, 30 s at 55°C, and 30 s at 72°C, preceded by an initial denaturing for 10 min at 95°C, and followed by a final extension for 5 min at 72°C. The residual primers were removed from the PCR product using the Agencourt AMpure XP PCR purification system (Beckman Coulter, Brea, CA, United States) following manufacturer’s instructions, aside from adjusting the template to AMpure volume ratio to 1:1 and repeating the ethanol wash step three times instead of two for maximum PCR product clean-up.

#### Sequence Analyses

The 16S rRNA gene libraries were sequenced using the Illumina MiSeq platform and 250 bp paired-end kits. Each sequencing run included a 15% PhiX spike. Sequence quality filtering, contig formation, and demultiplexing were performed using QIIME (Caporaso et al., 2010). We aligned sequences against the GreenGenes 16S rRNA gene reference database (v.13_8; DeSantis et al., 2006); identified chimeras using CHIMERASLAYER (Haas et al., 2011); and removed chimeric sequences, singletons, and non-aligned sequences from our dataset. We assigned sequences to operational taxonomic units (OTUs) based on 97% sequence similarity, and assigned them to taxon affinities using the RDP Naïve Bayesian classifier (Wang et al., 2007). After assigning taxonomy, we identified archaeal, chloroplast, and mitochondrial sequences, and removed these non-target sequences from the dataset. Prior to alpha diversity analyses, we rarefied our samples to 10,000 sequences per sample (Supplementary Figure S1).

### Data Analyses

#### Richness and Evenness

Rarefaction was only used in richness and evenness index comparisons. Three alpha diversity parameters were calculated using the QIIME alpha_diversity.py script (Caporaso et al., 2010): richness (observed number of OTUs), evenness (Pielou’s *J*), and Simpson’s (1−*D*). We compared the three indices using a one-way ANOVA and assessed pairwise differences using a *post hoc* Tukey’s HSD test in R (R Core Team, 2018).

#### Variable Selection

We tested seven variables for their contributions to the variation in gut microbiota composition in various data subsets (Table 4). We considered three variables associated with host phylogeny (Genus, Species, and Subspecies for dunlin only), and three variables associated with sampling site and habitat (Biome, Habitat, and Site), and sampling Year. Sampling sites were divided into low Arctic and sub-Arctic in our Biome variable based on their latitude. Bird species were assigned to one of four habitat categories that they nested in during the breeding season: Terrestrial (T), terrestrial/mesic (TM), mesic (M), and aquatic (A) (Cunningham et al., 2016; K. Grond and J. A. Cunningham, personal observations).

**TABLE 4:** Host and site variables used to test for contributions to variation in gut microbiota composition in fecal samples from Arctic-breeding shorebirds collected from 2011 to 2014.

**Variable**	**Description**	**Levels**	**Used in data subset^∗∗∗^**
Site	Sampling site	Cold Bay, Yukon Delta, Nome, Cape Krusenstern, Utqiagvik, Ikpikpuk River, Colville River, Canning River, Mackenzie River^∗^	I–VII
Biome	Broad habitat category of sampling locations	Low Arctic, sub-Arctic	I, IV–VII
Habitat	Local habitat used by host species	Terrestrial (T), terrestrial/mesic (TM), mesic (M), and aquatic (A)	I–IV
Genus	Host genus	*Pluvialis*, *Calidris*, *Limnodromus*, *Phalaropus*	I–III
Species	Host species	American golden plover, long-billed dowitcher, pectoral sandpiper, semipalmated sandpiper, western sandpiper, dunlin, red-necked phalarope, red phalarope^∗∗^	I–V
Subspecies	Subspecies of dunlin	*Calidris alpina arcticola*, *Calidris alpine pacifica*	VI

*^∗^Cold Bay and Yukon Delta were only used in the dunlin subset, since they only contained one shorebird species. ^∗∗^American golden plover and long-billed dowitcher only occurred at Utqiagvik, and are therefore only used to address inter-specific variation in microbial communities. ^∗∗∗^Subset composition can be found in the section “Data Analysis” of the section “Materials and Methods.”*

#### Data Sets

To assess variables affecting gut microbiota composition in shorebirds at different environment and host-relatedness levels, we considered seven different subsets of our samples for analyses:

(I) All fecal samples of breeding shorebirds (*n* = 306 individuals). We excluded samples from Cold Bay and the Yukon Delta, as only these two sites contained a single species. In addition, we excluded two shorebird species that were only found at a single site (American golden plover and long-billed dowitcher).
(II) Low Arctic sites (*n* = 242). Samples from Utqiagvik (formerly known as barrow), Ikpikpuk River (IKRI), Colville River, Canning River, Mackenzie River Delta (MARI). We classified sites situated above 68° latitude as low Arctic.
(III) Sub-Arctic sites (*n* = 90). Samples from Nome and Cape Krusenstern. Sites situated below 68° latitude were classified as sub-Arctic. We excluded samples from Cold Bay and the Yukon Delta, as only dunlin were sampled at these sites.
(IV) Calidrids (*n* = 257). All samples from four species in the Genus *Calidris*: Pectoral sandpiper (*n* = 16), dunlin (*n* = 71), semipalmated sandpiper (*n* = 128), and Western sandpiper (*n* = 42). Pectoral sandpipers are different from the other Calidrids in that they are promiscuous over a large range, whereas the other three species are socially monogamous with strong site fidelity (Kempenaers and Valcu, 2017; Weiser et al., 2018). We excluded samples from Cold Bay and the Yukon Delta, as only dunlin were sampled at these sites.
(V) Phalaropes (*n* = 30). Samples from two species in the Genus *Phalaropus*: red phalarope (*n* = 16) and red-necked phalarope (*n* = 14) at three sites where both species occurred: Canning River, Colville River, and IKRI. Phalaropes breed in terrestrial habitats but are pelagic during the non-breeding season.
(VI) Dunlin (*n* = 106). Dunlin samples included two subspecies: *C. alpina arcticola* (*n* = 58) sampled at Utqiagvik, IKRI, Colville River, and Canning River, and *C. alpina pacifica* (*n* = 48) sampled at Cold Bay, Yukon Delta, and Cape Krusenstern (South to North). *C. a. arcticola* spends the non-breeding season in East Asia, while *C. a. pacifica* winters in the Pacific Northwest during this period.
(VII) Semipalmated sandpipers (SESA) (*n* = 128). SESA were widely distributed and sampled at six sites: Nome, Cape Krusenstern, Colville River, IKRI, Canning River, and MARI (West to East). Western populations spend the non-breeding season on the Pacific coast of South America, whereas eastern populations use north-eastern South American coasts (Brown et al., 2017).

#### Variable Significance and Contribution

Statistical analyses were conducted in R (version 3.4.3; R Core Team, 2018). We generated weighted and unweighted UNIFRAC distance matrices (UDM) for microbial communities at an OTU level (Lozupone and Knight, 2005). Weighted UDMs take OTU abundance into account, whereas unweighted UDMs only account for presence/absence of OTUs within a sample. We tested for significance of the selected variables using the adonis function in the “vegan” package in R in weighted and unweighted UDMs. After identifying factors that significantly contributed to the variation in our datasets, we determined their relative contributions with a multifactorial permutational multivariate analyses of variance (PERMANOVA), using the adonis function in the “vegan” package in R (Oksanen et al., 2018). We randomly permuted the order of the variables in our multifactorial PERMANOVA, to test whether variable order affected significance and relative contribution.

To visualize differences among microbial communities within our datasets, we applied non-metric multidimensional scaling (NMDS) of Bray–Curtis distance matrices using the metaMDS function with *k* = 2 dimensions in the “vegan” package. In addition, to assess contribution of our explanatory variables to the variation in the NMDS, we fitted the variables to the ordination using the envfit function in the “vegan” package.

## Results

### Bacterial Composition of the Shorebird Gut Microbiota

#### Richness and Evenness

**FIGURE 2:**
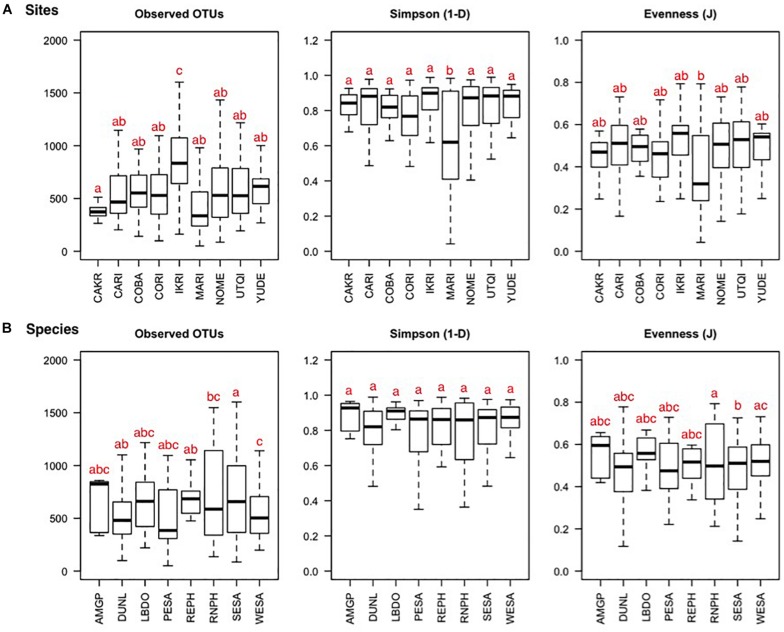
Bacterial OTU richness, diversity, and evenness depicted per sampling site **(A)** and host species **(B)** of fecal samples collected from eight species of shorebird at nine Arctic and sub-Arctic breeding sites. The black line represents the median, 25 and 75% quartiles are shown in the boxes, and 90% confidence intervals are enclosed within the whiskers. Letters represent pair-wise significance (Tukey’s HSD), with the different letters representing significant differences at α = 0.05. Site and host species abbreviations can be found in Tables 1, 2.

From the 375 fecal samples, we detected 34 bacterial Phyla, and a total of 24,944 unique OTUs. Richness indices are shown ±standard error (SE). 0.1% of sequences could not be classified on a Phylum level. On average, we detected 12.0 ± 0.18 SE Phyla and 684.9 ± 16.1 SE OTUs per fecal sample. Richness, diversity, and evenness indices differed among the nine field sites (Figure 2A; observed OTUs: *F*_(__8__,__366__)_ = 10.4, *p* < 0.001; Simpson 1−*D*: *F*_(__8__,__366__)_ = 2.96, *p* = 0.003; Evenness *J*: *F*_(__8__,__366__)_ = 2.53, *p* = 0.011). The differences were driven by a higher OTU richness at the IKRI site (Tukey’s HSD, observed OTUs: *p* = 0.007 ± 0.005 SE) and lower richness and evenness at the MARI (Tukey’s HSD, Simpsons 1−*D*: *p* = 0.013 ± 0.011; Evenness *J*: *p* = 0.001). Number of OTUs, but not diversity and evenness, differed among shorebird species (Figure 2B; observed OTUs: *F*_(__7__,__367__)_ = 5.59, *p* < 0.001; Simpson 1−*D*: *F*_(__7__,__367__)_ = 0.88, *p* = 0.523; Evenness *J*: *F*_(__7__,__367__)_ = 1.36, *p* = 0.220). Western Sandpipers (WESA) had a slightly lower number of OTUs, while SESA had a higher number of OTUs compared to all other species.

#### Taxon Diversity

**FIGURE 3:**
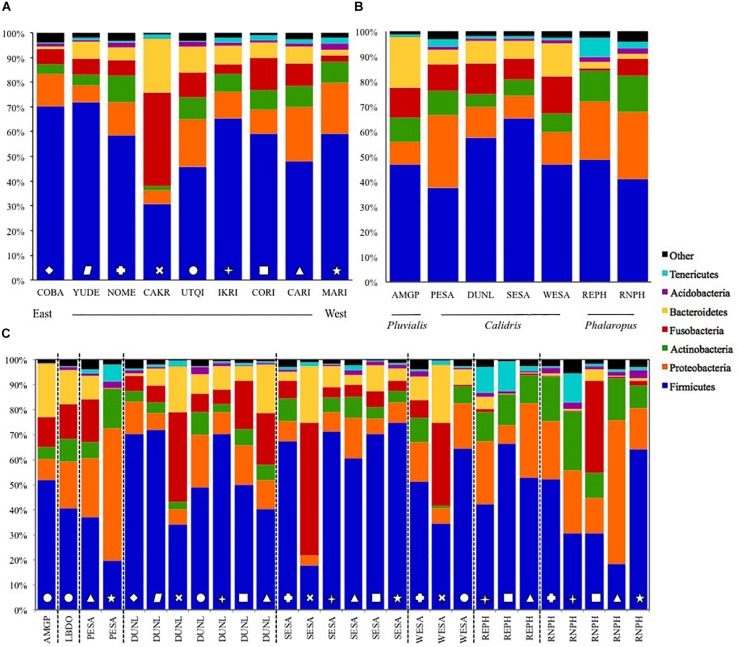
Relative abundance of fecal microbial Phyla collected from Arctic-breeding shorebirds from 2011 to 2014 averaged per site **(A)** and per species **(B)**, and with site and species combined. Sites are listed from west to east, and site and species abbreviations can be found in Tables 1, 2. Symbols on bars in panel **(C)** represent samples from different sites, as identified in panel **(A)**.

The five dominant bacterial Phyla in our samples were: Firmicutes (55.4 ± 1.4%), Proteobacteria (13.8 ± 0.9%), Fusobacteria (10.2 ± 0.9%), Bacteroidetes (8.1 ± 0.7%), and Actinobacteria (7.5 ± 0.5%; Figures 1, 3). The two most abundant Classes within the Firmicutes were the Bacilli (43.0%) and Clostridia (14.7%; Supplementary Figure S2). Bacilli were dominated by species within the Order Lactobacillales, and the Genus *Lactobacillus*.

Fusobacteria were the third dominant Phylum in our samples, but the relatively high abundance was driven by samples collected at Cape Krusenstern (Figures 1, 3A). Gut microbiota of shorebirds sampled at Cape Krusenstern (Fusobacteria: 31.2 ± 3.6%) included two genera that comprised >98% of all Fusobacteria: *Fusobacterium* spp. (60.5%) and *Cetobacterium* spp. (37.5%; Supplementary Figure S3). The relative abundance of these two genera within the Fusobacteria was similar among most sites (Supplementary Figures S2, S4).

#### Core Microbiota

We defined the core microbiota of shorebirds as the subset of OTUs that were present in >50% of the samples (Unterseher et al., 2011). Core microbiota included 67 OTUs, or ca. 0.3% of all OTUs detected. Core OTUs differed among sites (envfit; *R*^2^ = 0.19, *p* < 0.001), but not among host species within a site (envfit; *R*^2^ = 0.03, *p* = 0.074). Overall, 66.3% of the core OTUs belonged to the Phylum Firmicutes, and specifically to the Order Lactobacillales (45.9%). The known avian bacterial pathogen *Clostridium colinum* comprised 4.5% of all sequences within the core microbiota. After Firmicutes, Fusobacteria were most abundant with 14.1%, followed by Bacteroidetes (8.9%) and Proteobacteria (7.4%).

### Drivers of the Variation in Shorebird Gut Microbiota

**FIGURE 4:**
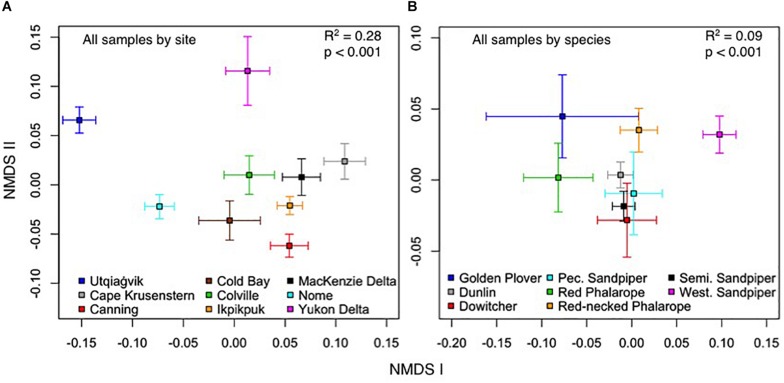
Non-metric multidimensional scaling of the contribution of: **(A)** sampling site and **(B)** host species to fecal microbiota composition of low Arctic- and sub-Arctic breeding shorebirds in 2011–2014. Squares represent centroids, and bars are standard error. *R*^2^- and *p*-values reflect overall significance of spatial patterning, although individual pairs of species may or may not be significantly different from each other.

Our full model, which included all explanatory variables (Table 4), explained 28.2% of the variation in the OTU composition of the gut microbiota of shorebirds during the breeding season. Independently, site explained the most variation in gut microbiota composition (*R*^2^ = 14.2%, *p* < 0.001, Table 5). Visual inspection of the NMDS showed that microbial communities were less similar if grouped by sampling site than by species, and that site explained more of the variation than species (Figure 4). Multifactorial PERMANOVA results showed Site as the dominant contributing variable in most datasets (I, III–VII; weighted UDM: *R*^2^ = 12.7–23.0%; Table 5), with exception of the Low Arctic (II), for which host species explained the most variation (*R*^2^ = 10.0%, *p* < 0.001).

**TABLE 5:** Multifactorial perMANOVA (adonis) tests for significance and relative contribution of seven environmental and host-related factors to variation in weighted and unweighted UniFrac Distance Matrices constructed from shorebird fecal communities.

		**Weighted UDM**		**Unweighted UDM**	
**Dataset**	**Variable**	***R*^2^**	***p* ≤**	***R*^2^**	***p* ≤**
All samples (I)	**Site**	**14.2**	**0.001**	**9.4**	**0.001**
	Biome	2.8	0.001	2.1	0.004
	Habitat	0.8	0.003	3.5	0.003
	Genus	4.2	0.002	2.6	0.001
	Species	6.2	0.001	4.2	0.001
	Year	1.0	0.001	0.4	0.013
Low Arctic^∗^ (II)	Site	7.2	0.001	5.8	0.001
	Habitat	1.2	0.004	1.2	0.001
	Genus	7.6	0.001	4.9	0.001
	**Species**	**10.0**	**0.001**	**7.1**	**0.001**
	Year	0.2	0.809	0.4	0.274
Sub-Arctic^∗^ (III)	**Site**	**23.0**	**0.001**	**10.4**	**0.001**
	Habitat	3.8	0.003	2.7	0.001
	Genus	5.6	0.001	2.5	0.006
	Species	4.8	0.031	4.2	0.014
	Year	1.6	0.052	1.1	0.036
Calidrids (IV)	**Site**	**16.6**	**0.001**	**10.6**	**0.001**
	Biome	3.3	0.061	2.5	0.003
	Species	2.2	0.004	1.9	0.002
	Year	0.9	0.007	0.6	0.004
Phalaropes (V)	**Site**	**14.6**	**0.007**	**10.1**	**0.001**
	Species	9.6	0.005	6.2	0.001
	Year	0.9	0.831	2.6	0.069
Dunlin (VI)	**Site**	**12.7**	**0.001**	**13.6**	**0.001**
	Biome	2.5	0.005	2.5	0.003
	Subspecies	2.5	0.007	2.5	0.001
	Year	0.6	0.629	0.9	0.407
Semipalmated sandpiper (VII)	**Site**	**20.1**	**0.001**	**13.6**	**0.001**
	Biome	3.9	0.001	4.1	0.001
	Year	2.5	0.007	0.8	0.189

*The highest *R*^2^ per dataset is bolded. ^∗^For classification of low and sub-Arctic sites, see the section “Data Analysis” in the section “Materials and Methods.”*

In our full dataset (I), Species also contributed significantly to the variation in the data (*R*^2^ = 6.2%, *p* = 0.001; Table 5), but to a lesser extent than Site. Changing the order of the variables in our PERMANOVA did not change our results. Weighted UDMs explained on average 2.2% more variation than the unweighted UDMs, which was likely due to low abundance OTUs being overrepresented in unweighted UDMs. In the dataset for Calidrids (IV), Site contributed 16.6% to the overall variation in microbial community, followed by 3.3% for Biome. The importance of Site as a driver of microbial variation was reflected in the single-species dataset for SESA (VII), for which Site contributed 20.1%, respectively. However, Site was less important as a driver in the single-species Dunlin dataset (*R*^2^ = 12.7%; VI), or the Phalarope dataset (*R*^2^ = 14.6%; V). We found a minimal but significant contribution of Dunlin subspecies (*C. alpina arcticola* and *C.a. pacifica*) to explaining the variation in microbiome composition (*F*_(__1__,__108__)_ = 1.605, *R*^2^ = 2.5%, *p* = 0.007).

## Discussion

We characterized the fecal microbiota of Arctic- and sub-Arctic-breeding shorebirds and investigated environmental and phylogenetic drivers of fecal microbial composition. Of the three environmental and three phylogeny-related factors we tested, breeding site (14.2%) contributed most to variation in fecal microbiota of breeding shorebirds, followed by host species (6.2%), the Genus of an individual sampled (4.2%), and the Biome the sampling sites were located in (2.8%). Habitat was a significant factor, but explained only 0.8% of the variation in fecal microbiota. Similar to other avian and mammalian studies (Waite and Taylor, 2014; Hird et al., 2015; Avena et al., 2016), most variation in fecal microbiota remained unexplained in our models (71.8%). High variability in low-abundance OTUs, which were possibly obtained from local environments, likely contributed to the unexplained variation we observed.

Our findings contrast with results from other comparative studies of avian microbiome studies (Waite and Taylor, 2014; Hird et al., 2015), which have concluded that host species largely determine microbial communities in the gut during migration and the non-breeding season. In our study, site was a driver of core gut microbiota, suggesting that microbial differences among Arctic breeding sites were driven by differences in common taxa, in contrast to the high number of peripheral, low abundance OTUs. Host species effects were not significant when investigating core microbiota, suggesting that all host species share a core microbiota, and that host effects are largely driven by rarer OTUs.

Another line of evidence that suggests site as an important driver of gut microbiota comes from observations of a close sequence match to *Clostridium colinum* in our samples (99% sequence similarity). We note that 16S amplicon sequencing is not suitable for high confidence species identification, but the high sequence similarity suggests a closely related species or strain. The bacterium was detected in 4.5% of all sequences within the core microbiota of shorebirds breeding in the nine Arctic and sub-Arctic sites. Interestingly, *C. colinum* (or close relative) comprised 37% of the microbiota found in adult red phalaropes at Utqiagvik, Alaska. We previously found that dunlin and red phalarope chicks sampled at Utqiagvik also had high relative abundances of *C. colinum* (Grond et al., 2017). Shorebird chicks likely acquire their microbiota from the local breeding environment, which concurs with our finding of breeding site as the most important driver of variation in microbiota for adult shorebirds (Grond et al., 2017). Also, a continued presence of *C. colinum* in adult microbiomes implies that either this initial inoculum is retained through life, or that adults are newly colonized by the bacteria every year at the breeding sites. It should be noted that *C. colinum* is considered an avian pathogen (Porter, 1998; Bildfell et al., 2001), but our data suggest that it may be part of the normal gut microbiota in migratory shorebirds.

We conducted the first project to investigate microbiome richness of breeding adult shorebirds. We detected ca. 25,000 unique OTUs in our 375 samples, but only 684.9 ± 16.1 OTUs per sample suggesting that each individual likely has a large proportion of unique – and possibly transient – community constituents. Bacterial richness of our samples was high compared to resident Neotropical birds and shorebirds (Hird et al., 2015; Risely et al., 2018), and even two times higher than found in other migratory shorebird species (Risely et al., 2017, 2018). The higher bacterial richness we detected could potentially be attributed to part of the annual cycle we sampled, but this is still speculative and should be investigated further.

A large diversity of gut microbiota could potentially benefit migratory birds by aiding in the digestion of local prey items or compete with novel pathogens found during migration (Quinn and Hamilton, 2012). Most studies that have identified host phylogeny as the main driver of microbial diversity have focused on non-migratory species of birds. In contrast, Lewis et al. (2016) found evidence that environment had a greater effect on gut communities than host species in migratory passerines (Lewis et al., 2016). Migratory birds are exposed to diverse environments during their annual cycle, potentially resulting in highly dynamic gut microbiota throughout the year. Diet of migratory birds can vary widely throughout the year. For example, several species of shorebirds switch from terrestrial arthropods during the breeding season to diets that consist of marine copepods, shellfish, diatoms, and bacterial biofilms during the non-breeding season (Quinn and Hamilton, 2012; Jardine et al., 2015).

Migratory birds can also harbor more pathogens, and have a higher infection intensity compared to resident bird species (Koprivnikar and Leung, 2015; Clark et al., 2016; Leung et al., 2016). It is possible that local microorganisms that incorporate gut microbiota could be better adapted to outcompete local pathogens and thus would indirectly be beneficial to the host, but no studies have investigated this hypothesis. Arctic environments often have lower microbial richness than low latitude sites (Fuhrman et al., 2008; Sul et al., 2013; Andam et al., 2016). We did detect a significant effect of latitude among our nine sites on the microbial richness in shorebird guts we studied, but this could be a result of the limited latitudinal range our sites were in.

### Microbiome Composition

Over half of OTUs detected in Arctic-breeding shorebirds belonged to the phylum Firmicutes. Firmicutes are consistently observed as a dominant phylum in wild birds (Grond et al., 2018), and these microorganisms are known to be involved in carbohydrate fermentation. From the Firmicutes detected, 43% belonged to the Class Bacilli. The closest relative within the Bacilli at a species level was *Lactobacillus ruminis* (99.3% sequence similarity), a common inhabitant of the gut environment in mammals and birds (O’Donnell et al., 2015; Rossi et al., 2016). The function of both *L. ruminis* and *C. colinum* in shorebirds is not known, but the high abundance in nesting birds with no symptoms of disease could indicate a commensal or beneficial role.

On average, relative abundance of Proteobacteria was low in shorebirds during the breeding season (13%), compared to during migration (55 and ∼20–60%; Grond et al., 2014; Risely et al., 2018), or the non-breeding season (35%; Risely et al., 2017). The function of Proteobacteria in the avian gut microbiome is not yet known (Grond et al., 2018), which makes it challenging to attribute the large differences in abundance to changing host requirements during different parts of the annual cycle. Also, the relatively large influence of environment on the shorebird microbiome we showed could indicate that environmental Proteobacteria abundances are site-specific. However, relative abundance of Proteobacteria in gut microbiomes from shorebirds sampled at Utqiagvik was 21%, while environmental Proteobacteria averaged 36% at this site in a previous study (Grond et al., 2017), suggesting some filtering by the host.

### Site and Species-Specific Findings

One interesting observation was the high relative abundance of Fusobacteria across all 34 individuals sampled at the Cape Krusenstern site. Fusobacteria are Gram-negative, non-spore forming bacteria and common members of the gastrointestinal microbiota in birds (Bennett et al., 2013; Dewar et al., 2014; Hird et al., 2015; Barbosa et al., 2016). *Fusobacterium* and *Cetobacteria* have been previously detected in shorebirds (Grond et al., 2014, 2017; Ryu et al., 2014). Cape Krusenstern is located ca. 332 km from the Nome site, where the relative abundance of Fusobacteria was lower and comparable to our other sites. In contrast to other sites, shorebirds at Cape Krusenstern foraged on mainly saline and brackish mud flats (M. L. Boldenow, personal communication). *Cetobacteria* are most commonly isolated from freshwater fishes (Tsuchiya et al., 2008; Larsen et al., 2014; Liu et al., 2016), but have also been detected in guts of sea mammals (Foster et al., 1995). A high abundance of Fusobacteria in shorebirds at Cape Krusenstern may thus result from local differences in foraging site and diet-associated microbiota.

Our NMDS showed substantial overlap in gut microbiota among most shorebird species, with the exception of WESA (Figure 4). WESA share breeding and non-breeding sites with SESA, but differ in diet during the non-breeding season. WESA have specialized bill and tongue morphology and feed on 40–60% surficial intertidal biofilm, comprised of microphytobenthos and bacteria (Kuwae et al., 2008; Jardine et al., 2015). Dunlin are also reported to forage on biofilm, but biofilm was reported to contribute only 2–14% to their daily energy expenditure (Kuwae et al., 2012). The high proportion of bacteria in the biofilm diet may carry over to affect the gut microbiome of the WESA during the breeding season, explaining the distinct communities. Behavioral observations and stomach content analyses would allow us to investigate the contribution of biofilm feeding on the gut microbiota dynamics of WESA.

We showed that of the variation explained by our models, breeding site was the dominant factor contributing to variation in gut microbiomes of migratory shorebirds. However, our models still explained only a relatively small fraction of the variability in gut microbiota, suggesting that there are other important drivers of shorebird microbiota (e.g., diet), or that microbial communities are highly dynamic within the shorebird gastro-intestinal tract. Also, to determine whether contributing factors and gut microbial composition are stable or dynamic during the annual cycle, we suggest extended sampling of migratory shorebirds elsewhere in their geographic range and at other stages of the annual cycle. Our study provided baseline data on the gut microbiomes of Arctic-breeding shorebirds, which could provide a baseline to compare future studies to. The rapid change in climate observed in the Arctic could impact shorebird microbiomes through bottom up effects, and our data will allow for future confirmation of such microbiome shifts.

## Data Availability Statement

The sequences and metadata supporting the conclusions of this article are available at Figshare https://doi.org/10.6084/m9.figshare.5885002.v1.

## Ethics Statement

The animal study was reviewed and approved by the Kansas State University Institutional Animal Care and Use Committee.

## Author Contributions

KG, RL, and BS contributed to the conception and design of the study. KG, RL, RB, MB, SB, BC, JC, AD, SF, BH, SK, EK, JL, LP-D, JR, and BS contributed to the fieldwork. KG, JS, AJ, RL, and BS contributed to the acquisition, analysis, and interpretation of the data. KG contributed to the writing of the manuscript. KG, JS, RL, AJ, RB, MB, SB, BC, JC, AD, SF, BH, SK, EK, JL, LP-D, JR, and BS contributed to the editing of the manuscript.

## Conflict of Interest

The authors declare that the research was conducted in the absence of any commercial or financial relationships that could be construed as a potential conflict of interest.
