# Supplementary figures and images for: Composition and Drivers of Gut Microbial Communities in Arctic-Breeding Shorebirds
Source: Front Microbiol. 2019 Oct 9;10:2258. doi: 10.3389/fmicb.2019.02258 (PMC6795060; doi:10.3389/fmicb.2019.02258)

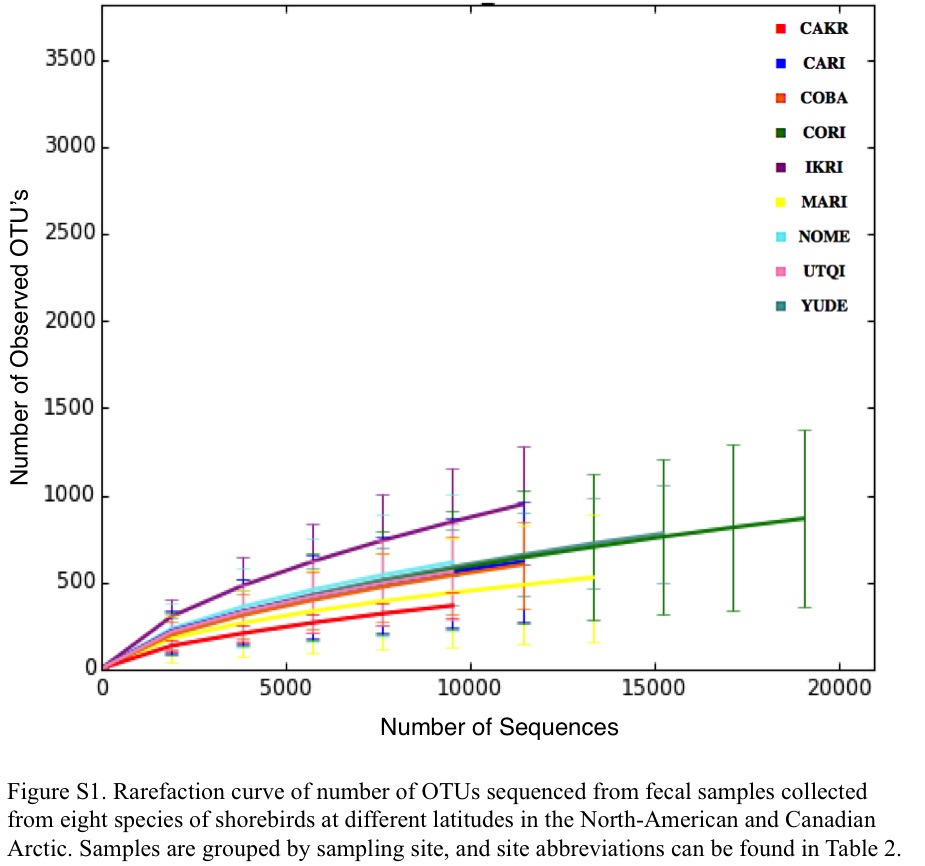

Supplement: Supplementary file 1 [file Image_1.JPEG]

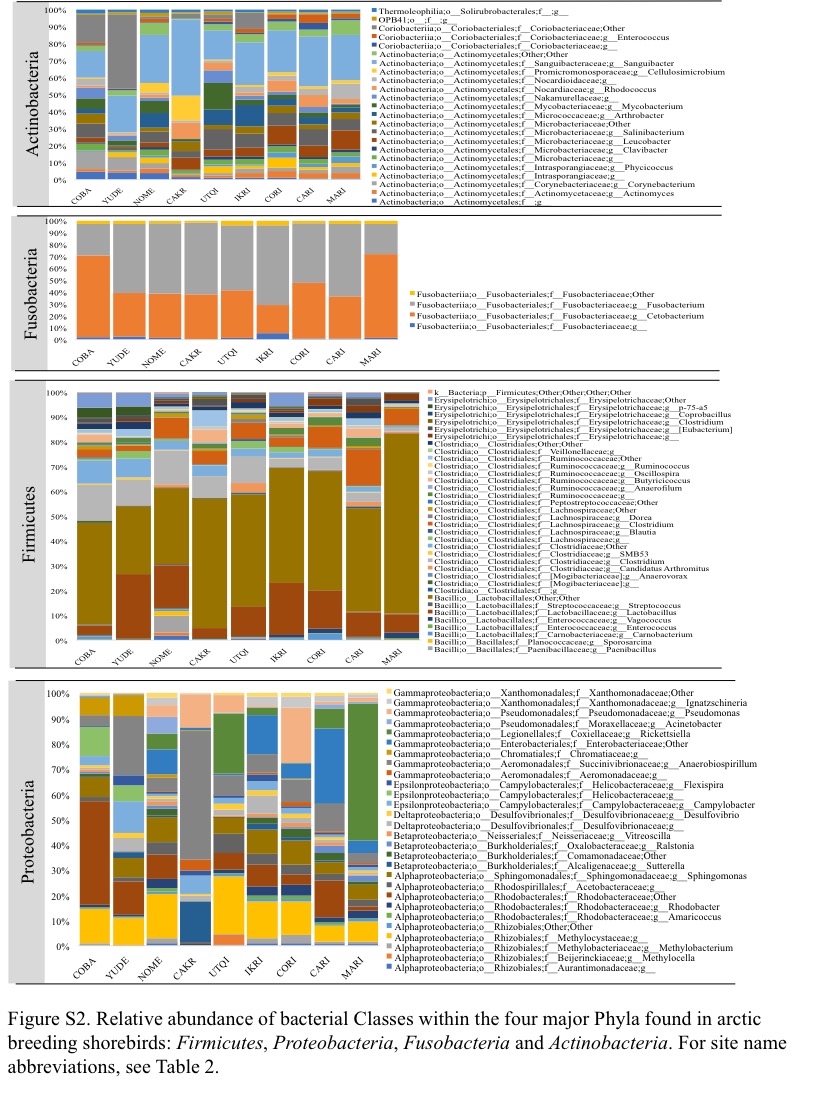

Supplement: Supplementary file 2 [file Image_2.jpg]

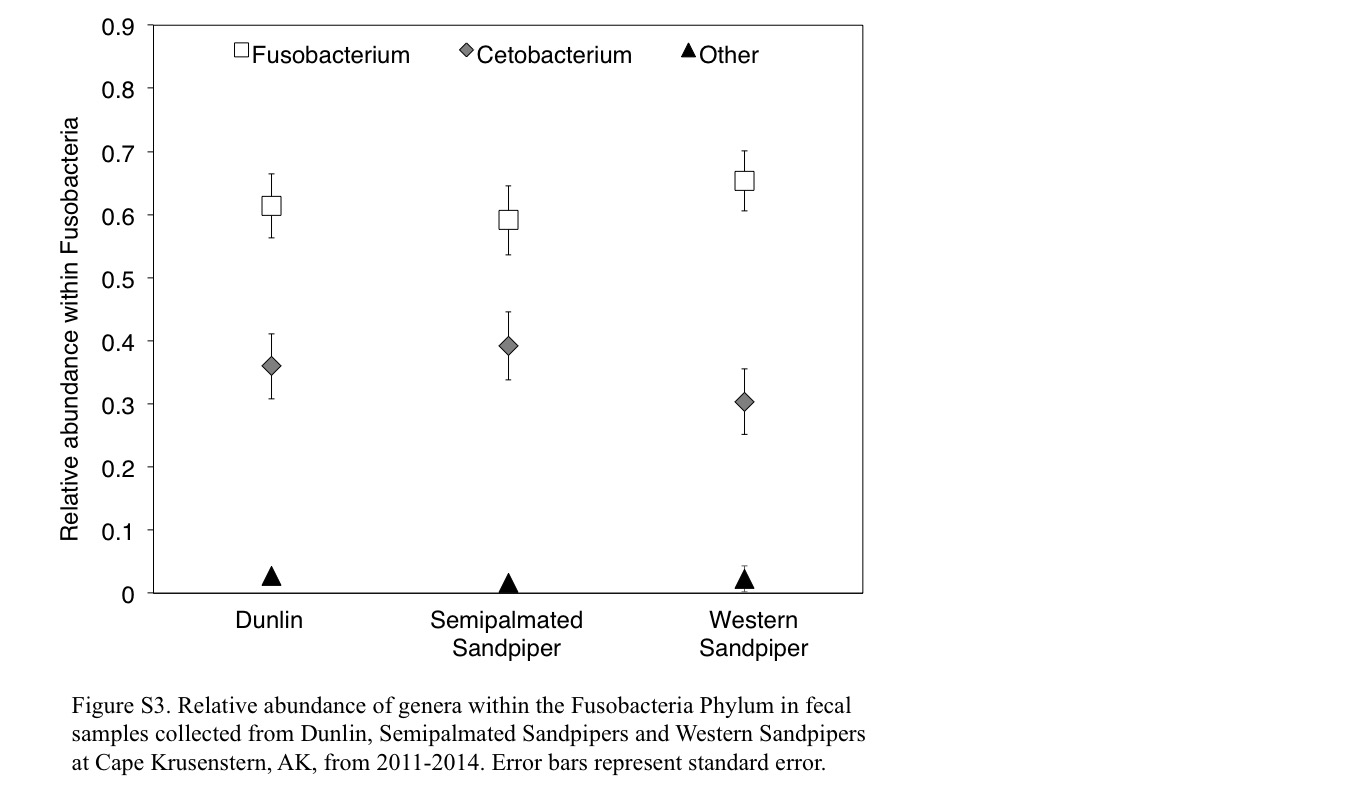

Supplement: Supplementary file 3 [file Image_3.JPEG]

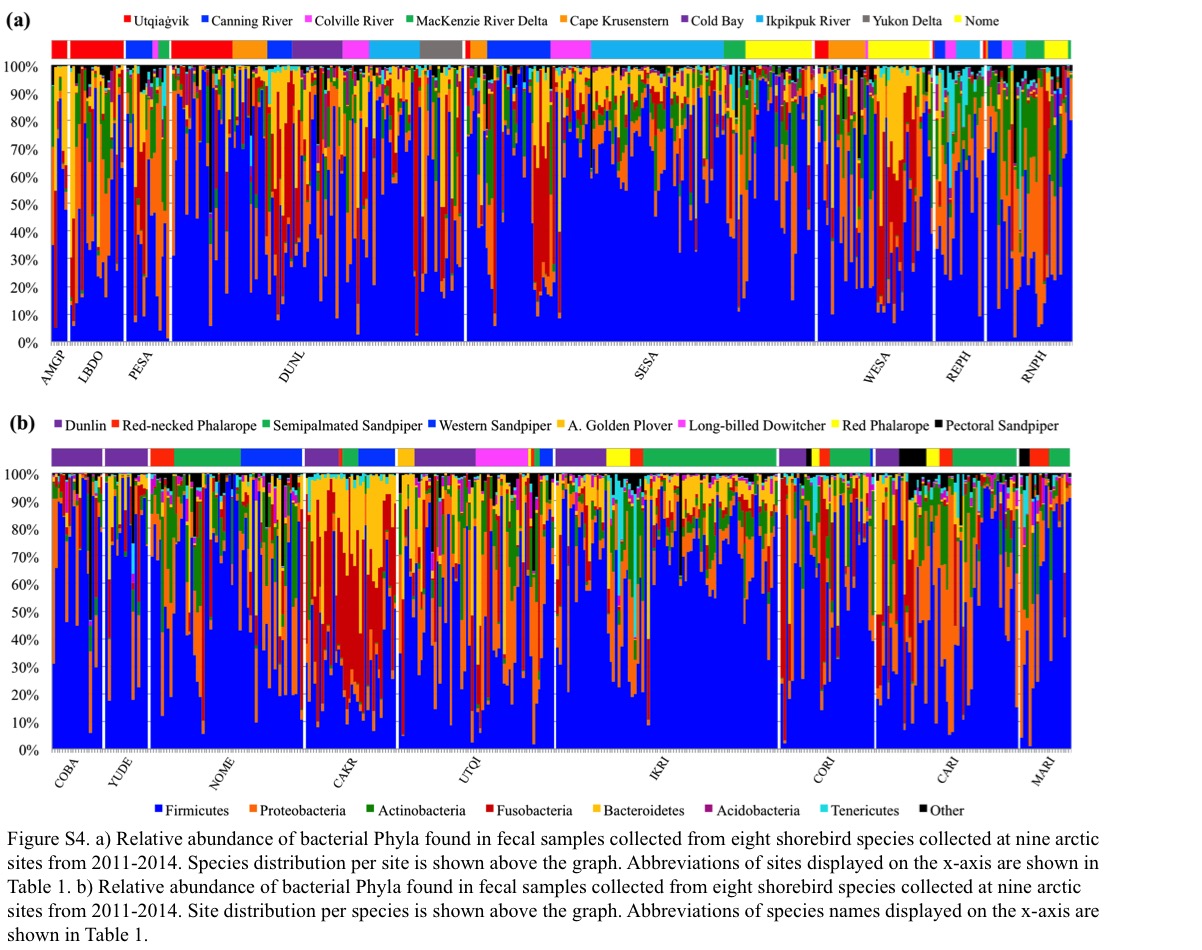

Supplement: Supplementary file 4 [file Image_4.JPEG]
